# A historical analysis of herpes simplex virus promoter activation *in vivo* reveals distinct populations of latently infected neurones

**DOI:** 10.1099/vir.0.2008/005066-0

**Published:** 2008-12

**Authors:** João T. Proença, Heather M. Coleman, Viv Connor, Douglas J. Winton, Stacey Efstathiou

**Affiliations:** 1 Division of Virology, Department of Pathology, University of Cambridge, Tennis Court Road, Cambridge CB2 1QP, UK; 2 Cancer Research UK Cambridge Research Institute, Li Ka Shing Centre, Robinson Way, Cambridge CB2 0RE, UK

## Abstract

Herpes simplex virus type 1 (HSV-1) has the capacity to establish a life-long latent infection in sensory neurones and also to periodically reactivate from these cells. Since mutant viruses defective for immediate-early (IE) expression retain the capacity for latency establishment it is widely assumed that latency is the consequence of a block in IE gene expression. However, it is not clear whether viral gene expression can precede latency establishment following wild-type virus infection. In order to address this question we have utilized a reporter mouse model system to facilitate a historical analysis of viral promoter activation *in vivo*. This system utilizes recombinant viruses expressing Cre recombinase under the control of different viral promoters and the Cre reporter mouse strain ROSA26R. In this model, viral promoter-driven Cre recombinase mediates a permanent genetic change, resulting in reporter gene activation and permanent marking of latently infected cells. The analyses of HSV-1 recombinants containing human cytomegalovirus major immediate-early, ICP0, gC or latency-associated transcript promoters linked to Cre recombinase in this system have revealed the existence of a population of neurones that have experienced IE promoter activation prior to the establishment of latency.

## INTRODUCTION

Herpes simplex virus type 1 (HSV-1) is a neurotropic alphaherpesvirus that establishes latency in sensory neurones innervating the site of primary infection. During latency, lytic virus gene expression is repressed and transcription is restricted to the latency-associated transcripts (LATs). LATs comprise a series of stable introns of 2.0 and 1.5 kbp, termed major LATs, which are spliced from a less abundant 8.3 kbp primary transcript termed minor LAT (reviewed by Wagner & Bloom, 1997). Major LATs accumulate within neuronal nuclei and provide a useful marker for the detection of latently infected neurones. However, estimates of the number of neurones harbouring virus DNA by *in situ* PCR (Maggioncalda *et al.*, 1996; Mehta *et al.*, 1995), laser capture microdissection (Chen *et al.*, 2002; Wang *et al.*, 2005) and direct analyses of dispersed neurones (Sawtell, 1997) indicate that LATs are transcribed in only a fraction (5–33 %) of all latently infected cells. The non-uniform nature of HSV latency is also apparent with respect to the amount of virus DNA within individual neurones, which can range from 1 to 100 copies of virus DNA in the majority of cells with rare neurones containing more than 1000 copies (Sawtell, 1997). The significance of this heterogeneity is unclear although high latent genome copy number may be a predisposing factor for virus reactivation (Sawtell *et al.*, 1998). Whether the high genome loads detected during latency are the result of direct loading from the periphery (Thompson & Sawtell, 2000) or the consequence of limited virus gene expression prior to the establishment of latency (Slobedman *et al.*, 1994) is currently unclear. Despite the fundamental nature of the question, it has not been possible to determine whether virus gene expression can occur prior to the establishment of latency using conventional techniques that rely on the detection of lytic cycle gene products at specific time points, since these promoters are efficiently silenced during latency. Thus, whilst recombinant viruses expressing reporter genes under lytic cycle promoter control are useful tools in the study of viral gene expression *in vivo* (Lachmann *et al.*, 1999; Smith *et al.*, 2000; Thompson *et al.*, 2003) this approach cannot be used to determine whether neurones experiencing lytic cycle promoter activity subsequently abort this pathway prior to the establishment of latency.

In order to directly address this issue we have utilized ROSA26R reporter mice (Soriano, 1999) containing a *LacZ* reporter gene whose expression is only induced following Cre-mediated recombination. Thus, following Cre-recombinase expression, cells are permanently genetically marked and can be identified by virtue of stable reporter gene expression. In this study, reporter mice have been used in conjunction with HSV-1 recombinants expressing Cre recombinase under latent or lytic cycle promoter control to determine whether viral promoter activity is compatible with cell survival and the establishment of latency. This system has revealed that lytic cycle promoter activation can precede latency establishment in a subpopulation of latently infected neurones *in vivo*.

## METHODS

### Cells and viruses.

All viruses are derived from HSV-1 strain SC16 (Hill *et al.*, 1975). Viruses were propagated and assayed on BHK cells unless stated otherwise. Cell lines were grown in Glasgow's modified Eagles medium (GMEM) supplemented with 10 % fetal calf serum (FCS) and 10 % tryptose phosphate broth. SUA cells are a Cre reporter Vero-derived cell line (Rinaldi *et al.*, 1999).

### Plasmids.

pHD5 is a plasmid designed to allow recombination of foreign sequences into the *US5* (gJ) gene of HSV-1 (Balan *et al.*, 1994). pGS403 (Smith & Enquist, 2000) is a pBluescript KSII-based plasmid containing the human cytomegalovirus (HCMV) major immediate-early promoter (MIEP) driving expression of Cre recombinase. pGS403n was derived from pGS403, but contains a single base pair C→G mutation at position 3597, creating an *Nco*I restriction site in the initiating ATG codon of the *Cre* gene. This mutation was created using a Stratagene QuickChange Site-Directed Mutagenesis kit. pHD5-CMVCre contains the Cre expressing cassette derived from pGS403 inserted into the *US5* gene in pHD5. To achieve this, pGS403 was digested with *Sal*I and *Sac*II and the resulting 2.6 kbp fragment end-repaired and cloned into the *Eco*RV site of pHD5. pHD5-ICP0Cre contains the HSV-1 ICP0 promoter (−818 to position +150 with respect to the ICP0 transcription start site) driving Cre recombinase in the US5 region. This plasmid was created by substituting the *β*-galactosidase (*β-Gal*) gene from pHD5110nt*β*Gal (Lachmann *et al.*, 1999) with the Cre expression cassette. The *β-Gal* gene was excised by digestion with *Bam*HI and substituted with an end-repaired *Hin*dIII–*Xho*I fragment from pGS403 containing Cre. pSLAT1 (Lachmann & Efstathiou, 1997) contains a 4.6 kb *Pst*I–*Bam*HI fragment encompassing major LATs, derived from HSV-1 SC16 *Bam*HI B (HSV-1 118867–123460) cloned into pBluescript M132 (Stratagene). pSLAT1*βgeo* (Lachmann & Efstathiou, 1997) was derived from pSLAT1, but contains the encephalomyocarditis virus (EMCV) internal ribosome entry site (IRES) linked to a *LacZ*–*neoR* gene fusion (*βgeo*) cloned into a 168 bp *Hpa*I deletion (HSV-1 120301–120469). pSLATCre contains the Cre recombinase gene under LAT promoter control. pSLAT1*βgeo* was digested with *Bbv*CI, end-repaired and redigested with *Nco*I to excise the *βgeo* gene. pGS403n was digested with *Xho*I, end-repaired and redigested with *Nco*I, to excise the Cre gene, which was then cloned into *Nco*I/*Bbv*CI-digested pSLAT1*βgeo*. To generate pGS-gC-403, the HSV-1 gC promoter [−200 to +124 relative to the gC transcription start site (Weir & Narayanan, 1990) HSV-1 95968–96296], was amplified by PCR from the HSV-1 strain 17 *Kpn*I D fragment using the following primers: forward 5′-TTTGAGCTCGAATTCTTCTTCTCCGTACGCGCTG-3′ and reverse 5′-TTTAAGCTTCCTCGCGAGGGATCGGCCTA-3′. These primers introduced novel *Sac*I and *Eco*RI restriction sites upstream of the gC promoter and a *Hin*dIII site was inserted downstream. The PCR fragment corresponding to the gC promoter was digested with *Sac*I/*Hin*dIII and inserted into the *Sac*I/*Hin*dIII sites in pGS403 placing Cre under control of the gC promoter. pHD5-gCCre contains the gC promoter-driven Cre recombinase inserted into the US5 region of HSV-1. pGS-gC-403 was digested with *Eco*RI/*Kpn*I and the fragment containing the gC promoter Cre cassette cloned into *Eco*RI/*Kpn*I cleaved pHD5.

### Construction and characterization of recombinant viruses.

**Fig. 1. f1:**
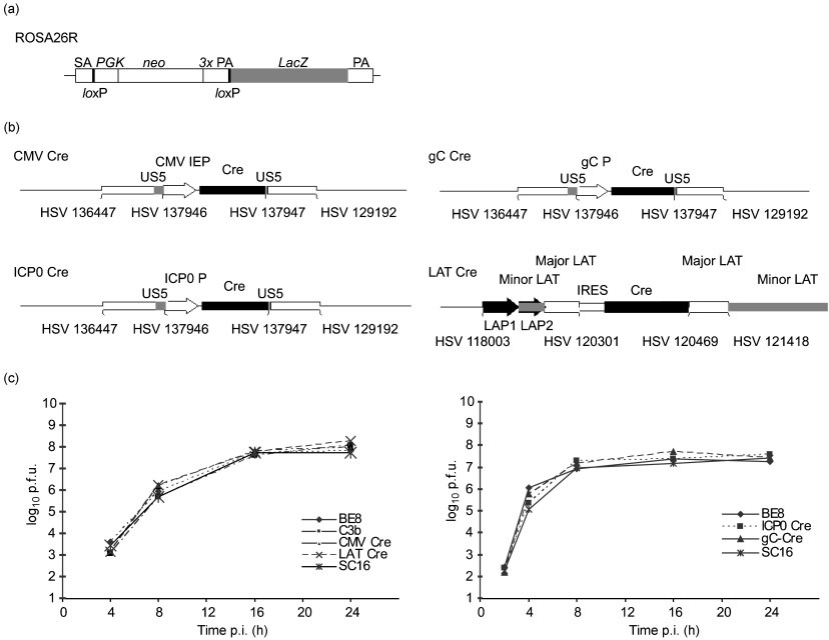
(a) Structure of the *ROSA26* locus in Cre reporter mice. The transgene contains a splice acceptor sequence (SA) upstream of a neomycin gene (*neo*) flanked by *lox*P sites and downstream of a *LacZ* gene. Following Cre recombination, the *neo* gene is removed and the *LacZ* gene is constitutively expressed by the *ROSA26* promoter. (b) Genomic structures of Cre expressing viruses: HSV CMV Cre, HSV ICP0 Cre and HSV gC Cre have a Cre expression cassette inserted in the non-essential *US5* region. This cassette contains the promoter of interest upstream of Cre recombinase. The Cre gene is fused to a nuclear localization signal and contains an intron 578 bp downstream of the transcription start site. The HSV LAT Cre virus contains an IRES-linked Cre gene inserted 1.5 kb downstream of the LAT transcription start site, placing Cre under LAT promoter control. (c) Single*-*step growth curves of recombinants, parental viruses and WT strain SC16 are from a single experiment performed in BHK cells.

HSV BE8 and HSV C3b contain an HCMV MIEP *LacZ* cassette inserted into the US5 locus and the 168 bp *Hpa*I deletion in the LAT region (HSV-1 120301–120469), respectively (Balan *et al.*, 1994; Lachmann & Efstathiou, 1997). HSV CMV Cre was constructed by co-transfecting pHD5-CMVCre and HSV BE8-infected cell DNA, producing a virus that contains the HCMV MIEP Cre recombinase cassette inserted at the US5 locus at the *Sst*I restriction site at genomic coordinate 137946. HSV LAT Cre was generated by co-transfecting pSLATCre and HSV C3b-infected cell DNA, thus inserting the Cre recombinase gene together with the EMCV-IRES 1.5 kb downstream of the LAT transcription start site. This virus thereby encodes Cre under the control of the LAT promoter. To produce HSV gC Cre, pHD5gCCre and HSV BE8-infected cell DNA were transfected into cells. The recombinant virus generated encodes Cre recombinase under gC promoter control at the US5 locus. The late kinetics of gC promoter-driven Cre transcription was confirmed by real-time PCR (data not shown). HSV ICP0 Cre was constructed by co-transfection of pHD5ICP0Cre and WT SC16-infected cell DNA. This recombinant virus contains the ICP0 promoter upstream of Cre recombinase inserted into the *Sst*I site at the US5 locus. In all cases, cell monolayers were harvested 3 days post-transfection and recombinant virus progeny selected based on failure to stain positive for *β*-Gal (HSV CMV Cre, HSV LAT Cre and HSV gC Cre) when compared with parental viruses (BE8 and C3b). HSV ICP0 Cre was selected based on its ability to switch on *β*-Gal expression in SUA cells. Virus recombinants were isolated and plaque purified by limiting dilution. Viral genomic structures (Fig. 1) were confirmed by restriction endonuclease digestion and Southern blot hybridization (data not shown). One-step growth curves were performed as described previously (Lachmann *et al.*, 1996).

*In vivo* studies used 7- to 8-week-old female BALB/c mice (Harlan). Mice under isoflurane anaesthesia were infected with 10^6^ p.f.u. of either WT or recombinant virus in 20 μl GMEM by scarification of the left ear. At various time points, groups of five mice were killed by CO_2_ asphyxiation. Left ear pinna and cervical dorsal root ganglia CII, CIII and CIV were dissected and placed into 1 ml medium for viral titrations and stored at –70 °C prior to assay. Ganglia for explant reactivation assays were placed in 1 ml GMEM containing 10 % FCS and incubated for 5 days at 37 °C in a gassed 5 % CO_2_ incubator. Explanted ganglia were then freeze–thaw and homogenized prior to assay. For the measurement of latent virus DNA loads, CII, CIII and CIV ganglia from five mice were pooled into TE (10 mM Tris, 1 mM EDTA pH 8.0) and frozen at −70 °C prior to DNA extraction. All animal experiments were performed under Home Office Project Licence 80/1806.

ROSA26R reporter mice (Soriano, 1999) were used for the *in vivo* characterization of HSV recombinants encoding Cre recombinase. Groups of adult mice (>8 weeks of age) that differed in age by less than 12 days were infected with 2×10^6^ p.f.u. of virus by scarification of the left ear. At various times after infection mice were killed and CII, CIII and CIV cervical ganglia pooled and fixed on ice for 1 h in 4 % paraformaldehyde in PBS and stained for X-Gal as described previously (Lachmann & Efstathiou, 1997) or embedded in paraffin for *in situ* hybridization analyses by using major LAT-specific digoxigenin-labelled probes (Arthur *et al.*, 1993; Lachmann & Efstathiou, 1997).

### DNA extraction for quantitative real-time PCR.

Pooled CII, CIII and CIV sensory ganglia from five mice were homogenized and incubated in 0.5 % SDS, 50 μg proteinase K ml^−1^ in TE overnight at 37 °C. DNA samples were subjected to phenol/chloroform extraction followed by column purification using Qiagen PCR purification kit. Real-time PCR was performed as described previously (Coleman *et al.*, 2008) using adenine phosphoribosyltransferase (APRT) and ICP0 promoter-specific primer sets. APRT forward primer (nt 585–604), reverse primer (nt 706–688) (Dush *et al.*, 1985) and Taqman probe (nt 667–640). ICP0 primers as described in Coleman *et al.* (2008).

### Statistical analysis.

Statistical differences between the numbers of marked cells per sensory ganglia from mice sampled at different time points were determined by the Mann–Whitney test.

## RESULTS

### *In vitro* and *in vivo* characterization of HSV-1 based recombinants encoding Cre recombinase

**Fig. 2. f2:**
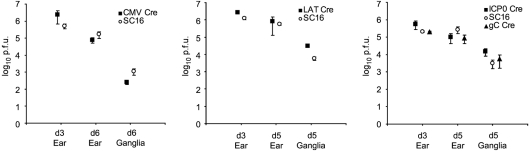
*In vivo* pathogenicity studies. Virus titres in ears and pooled CII, CIII and CIV ganglia of BALB/c mice sampled at the indicated time points. Data points represent average viral titres from five mice±sem for each recombinant and WT SC16. Each panel represents an independent experiment.

**Fig. 3. f3:**
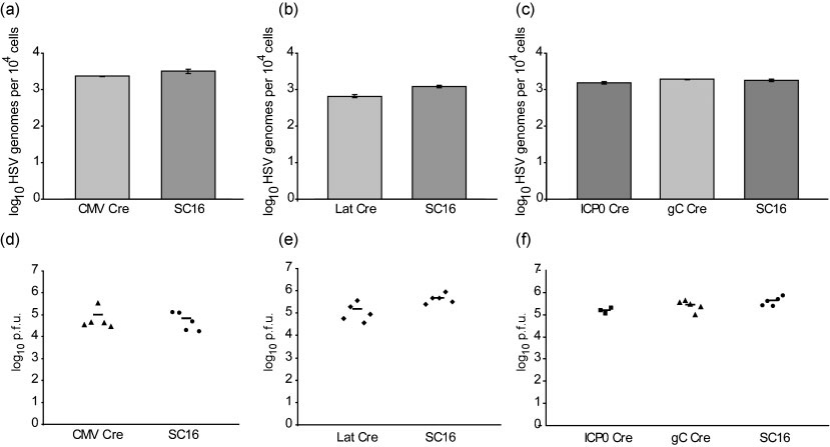
Latent DNA loads and explant reactivation capability of virus recombinants. (a, b, c) Latent DNA loads: real-time PCR was performed on DNA extracted from latently infected ganglia (CII, CIII and CIV pooled from five mice) using as targets ICP0 and APRT. Values represent the mean±sem of the numbers of the HSV genome copies per 10^4^ copies of APRT of three real-time PCRs. (d, e, f) Viral explants reactivation titres: CII, CIII and CIV ganglia from latently infected mice were explant cultured for 5 days and assayed for reactivated virus. Symbols represent viral titres detected per mouse and the bars are their mean.

Viruses expressing Cre recombinase under HCMV MIEP, LAT, ICP0 IE and gC late promoter control were constructed on an HSV-1 SC16 background as described in Methods. Schematic representations of the ROSA26R locus and recombinant virus structures are shown in Fig. 1(a, b). All recombinants replicated with wild-type (WT) kinetics *in vitro* (Fig. 1c[Fig f1]) and acute phase replication kinetics in the ears and CII, CIII and CIV sensory ganglia of mice revealed no obvious growth deficit of recombinant viruses (Fig. 2). Real-time PCR-based quantification of latent DNA loads in sensory ganglia and explant reactivation assays revealed that all recombinants established latency to WT levels (Fig. 3). These data indicate that the recombinant viruses are phenotypically indistinguishable from WT virus *in vivo* and are consistent with previous observations concerning the lack of detectable phenotypes of viruses carrying gene insertions 1.5 kb downstream of the LAT transcription start site (Lachmann & Efstathiou, 1997) or *US5* gene loci (Balan *et al.*, 1994; Thompson *et al.*, 2003).

### Reporter mice facilitate the marking of latently infected neurones following infection with HSV CMV Cre

**Fig. 4. f4:**
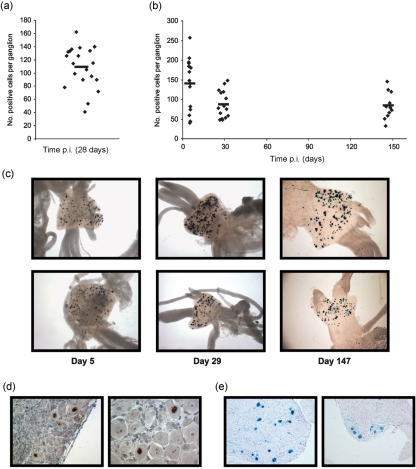
*In vivo* reporter gene activation by HSV CMV Cre. (a, b) Number of positive cells per ganglia from two independent experiments, each symbol represents a dorsal root ganglion and the bar represents the mean cell count. (c) Light micrographs of X-Gal stained sensory ganglia at 5, 29 and 147 days p.i. (d) LAT *in situ* hybridization of ganglionic sections sampled from mice 5 months p.i. revealed an average of 1.05 LAT-positive cells per ganglionic profile. (e) Sections of X-Gal stained ganglia from the same experimental group of mice, revealed an average of 9.7 *β*-Gal-positive cells per ganglionic profile.

We next determined whether HSV-1 recombinants encoding Cre recombinase could activate reporter gene expression in the nervous system of ROSA26R mice. Our initial attention focused on the HCMV MIEP Cre recombinant (HSV CMV Cre) since previous studies have shown that this powerful promoter is transiently active prior to the establishment of latency in cultured sensory neurones (Arthur *et al.*, 2001) and unlike HSV IE promoters, activation of the HCMV MIEP is not dependent on the HSV virion transactivator VP16. ROSA26R mice were infected with HSV CMV Cre and following resolution of acute infection, latently infected cervical dorsal root ganglia were stained for *LacZ* expression. In this mouse strain, expression of Cre recombinase mediates recombination and excision of the *lox*P flanked neomycin resistance gene located between the promoter and the reporter gene, resulting in constitutive *LacZ* expression. Of importance to these studies is the recognition that transient viral Cre recombinase expression results in the permanent genetic marking and identification of latently infected neurones. Infected mice (*n*=7) were sampled 28 days post-infection (p.i.) and pooled CII, III and IV sensory ganglia were examined for reporter gene expression. In contrast to the rapid shutdown of reporter gene expression in neurones both *in vitro* and *in vivo* following infection with recombinants containing an HCMV MIEP-driven reporter gene (Arthur *et al.*, 2001; Balliet *et al.*, 2007; Scarpini *et al.*, 2001; Smith *et al.*, 2000), CMV Cre-induced reporter gene expression was readily detected during latency in sensory ganglia [mean 108.89 *β*-Gal-positive neurones per ganglion±7.04 (sem)] of ROSA26R mice (Fig. 4a[Fig f4]). This result indicates that HCMV MIEP-driven Cre expression in the context of HSV-1 can mediate *lox*P recombination and subsequent reporter gene activation during the establishment of latency. In order to examine the stability of this population of marked cells, ROSA26R mice were infected and sampled at either 5, 29 or 147 days p.i. Examination of pooled sensory ganglia stained for *β*-Gal (Fig. 4b, c[Fig f4]) revealed positively stained cells during acute infection (day 5) with an average of 140.3±17 *β*-Gal-positive neurones per ganglion. At both 29 and 147 days p.i. similar numbers of *β*-Gal-positive neurones per ganglion were detected with mean counts of 87.3±9 and 83.9±10.2, respectively. The reduction in the number of marked cells observed between day 5 and latent time points is likely to reflect the death and loss of productively infected cells. The stability and number of marked neurones at latent time points suggests that HCMV MIEP-driven Cre expression results in the stable marking of a large cohort of latently infected cells. In order to determine the relationship between the number of reporter gene-positive neurones and neurones expressing LATs, pooled sensory ganglia were sampled from groups of five mice and either stained for *β*-Gal or processed for *in situ* detection of LATs (Fig. 4d, e[Fig f4]). Ganglia from mice sampled 5 months p.i. were sectioned and the number of *β*-Gal-positive or LAT-positive neuronal profiles per ganglionic section enumerated. *In situ* hybridization identified an average of 1.05 LAT-positive neuronal profiles per ganglionic section (*n*=46), whereas histochemical analyses revealed 9.7 *β*-Gal-positive neuronal profiles per ganglionic section (*n*=127). The number of *β*-Gal-positive profiles per ganglionic section was approximately ninefold greater than the number of LAT-positive neuronal profiles, indicating that Cre-mediated reporter gene activation is a more sensitive method by which to identify latently infected cells than conventional *in situ* detection of LATs. Given that independent studies have reported that *in situ* detection of LATs reveals approximately 1/3 of all neurones containing HSV DNA (Maggioncalda *et al.*, 1996; Mehta *et al.*, 1995), we conclude that reporter gene activation by HCMV MIEP Cre expression is likely to mark the majority of latently infected neurones *in vivo*.

Previous reports describing the detection of LATs in only a proportion of latently infected neurones may reflect the relative insensitivity of conventional hybridization methods to detect low-level LAT transcription (Ramakrishnan *et al.*, 1996) or the existence of a population of latently infected neurones in which the LAT promoter is inactive at a given time point. In order to distinguish between these possibilities we next examined a recombinant virus encoding Cre recombinase under LAT promoter control in order to determine the number of neurones that experience LAT promoter activity during the establishment of latency.

### Reporter mice facilitate the marking of a large reservoir of latently infected neurones following infection with HSV LAT Cre

**Fig. 5. f5:**
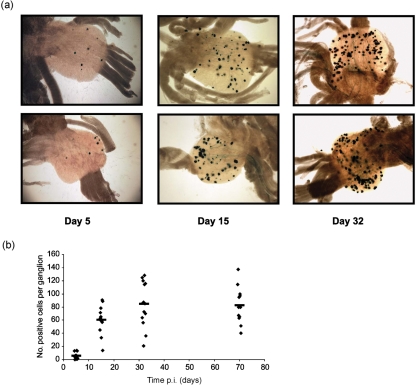
*In vivo* reporter gene activation by HSV LAT Cre virus. (a) Representative light micrographs of cervical ganglia stained with X-Gal at 5, 15 and 32 days p.i. (b) The number of *LacZ*-positive cells per ganglion at 5, 15, 32 and 70 days p.i. The symbols represent the number of positive cells in each ganglia and the bar represents the mean number of *β*-Gal-positive cells.

ROSA26R mice were infected with a recombinant virus expressing Cre recombinase under LAT promoter control. In this construct an IRES-linked Cre gene was inserted 1.5 kb downstream of the LAT transcription start site. Insertion of reporter genes at this locus has been shown previously to result in long-term neuronal transgene expression in the context of both WT virus (Lachmann & Efstathiou, 1997) and replication defective HSV-1 (Marshall *et al.*, 2000; Scarpini *et al.*, 2001). In contrast to the HCMV MIEP, which resulted in the marking of large numbers of neurones during the acute stage of infection, the HSV LAT Cre recombinant resulted in the marking of only a small number of neurones per ganglion (mean 5.6±1.9) (Fig. 5a, b[Fig f5]). Thus, the small number of neurones marked by the LAT promoter, most likely reflect the slow activation of this promoter during the early stages of latency establishment. By 15 days p.i., the number of marked cells had increased to a mean of 60±6.3 and thereafter stabilized with 84±8.5 and 82.4±8 marked cells per ganglion detected at 32 and 70 days p.i., respectively (Fig. 5a, b[Fig f5]).

### Infection of ROSA26R mice with HSV ICP0 Cre identifies a stable population of latently infected neurones during latency

**Fig. 6. f6:**
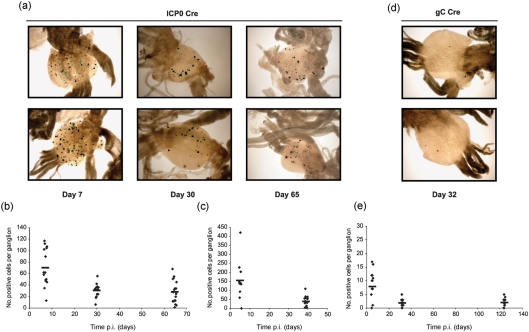
*In vivo* reporter gene activation by HSV ICP0 Cre and HSV gC Cre recombinant viruses. (a) Representative micrographs of histochemical detection of *β*-Gal in the dorsal root ganglia of reporter mice infected with HSV ICP0 Cre at 7, 30 and 65 days p.i. (b, c) Numbers of *LacZ*-positive cells per ganglion marked by HSV ICP0 Cre from two independent experiments at the specified time points. Symbols represent the number of positive cells in a single ganglion and the bars the mean number of *β*-Gal-positive cells at the indicated times. (d) Representative micrographs of histochemical detection of *β*-Gal in sensory ganglia of reporter mice infected with the HSV gC Cre recombinant at 32 days p.i. (e) Numbers of positive cells per ganglion at 5, 32 and 124 days p.i. with HSV gC Cre virus.

In order to determine whether HSV-1 IE ICP0 promoter activation during latency establishment is compatible with cell survival, we constructed an HSV recombinant encoding Cre under ICP0 promoter control. Previous studies have demonstrated that ICP0 promoter-reporter constructs accurately reflect the pattern of ICP0 protein expression from the endogenous gene during acute neuronal infection both *in vitro* (Arthur *et al.*, 2001) and *in vivo* (Thompson *et al.*, 2003). Furthermore, such promoter-reporter constructs are efficiently silenced during latency (Lachmann *et al.*, 1999; Shimeld *et al.*, 2001; Thompson *et al.*, 2003). ROSA26R mice infected with HSV ICP0 Cre were sampled at acute (day 7 p.i.) and latent (day 30 and 65 p.i.) time points. Sensory ganglia were pooled from five mice and stained for *β*-Gal expression (Fig. 6a[Fig f6]). At day 7 p.i., 70.2±8.4 *β*-Gal-positive neurones per ganglion were identified (Fig. 6b[Fig f6]). Since day 7 p.i. represents an acute stage of infection it was considered likely that these *β*-Gal-positive neurones corresponded to productively infected cells, which would be eliminated as the infection progressed. Significantly, the number of *β*-Gal-positive neurones per ganglion decreased to 30.1±3 at day 30 p.i. and remained at a relatively stable level thereafter with 28.1±4.6 neurones per ganglion labelled at 65 days p.i. (Fig. 6b[Fig f6]). The number of cells marked during latency was confirmed in an independent experiment, which identified 154±32.5 marked cells at the peak of acute infection (day 5 p.i.), and 37.9±7.7 marked neurones per ganglion at 39 days p.i. (Fig. 6c[Fig f6]). This result suggests that approximately 35 % of neurones marked by either HCMV IE or LAT promoter-driven Cre expression are likely to have experienced ICP0 promoter activation prior to the establishment of latency.

In contrast with HSV ICP0 Cre, infection of reporter mice with an HSV-1 recombinant expressing Cre under the control of the late gC promoter resulted in a low level of neuronal marking at the acute day 5 time point (7.8±1.5 *LacZ*-positive neurones per ganglion) (Fig. 6d, e[Fig f6]). Furthermore, an even lower level was observed at days 30 and 124 latent time points, which revealed 1.9±0.35 and 2±0.36 *β*-Gal-positive neurones per ganglion, respectively. These data indicate that late promoter activation of Cre expression is largely incompatible with cell survival.

## DISCUSSION

In this paper ROSA26R reporter mice have been used in conjunction with HSV-1 recombinants expressing Cre recombinase to genetically mark neuronal cells. This approach to studying HSV pathogenesis and latency has significant advantages over the use of viruses expressing reporter genes under the control of HSV-1 promoters since such recombinants only facilitate the examination of reporter gene expression in tissues at defined time points (Lachmann *et al.*, 1999; Smith *et al.*, 2000; Thompson *et al.*, 2003). This limits their utility in studies of pathogenesis since lytic cycle promoters are silenced during latency. Thus, the question of whether lytic cycle promoter activity can be tolerated by neurones prior to the establishment of latency remains largely unexplored.

Infection of reporter mice with HSV CMV Cre resulted in the efficient marking of sensory neurones that extended from the acute to latent phase of infection. The mean number of marked neurones per ganglion at the acute day 5 time point exceeded the number of cells marked at days 30 and 150 latent time points by 1.6-fold (*P*=0.031). The higher numbers detected during the acute stage is likely to be due to the marking of both acutely infected cells, which are destined to die as a result of productive infection, in addition to a cohort of cells that have experienced transient HCMV MIEP activation prior to the establishment of latency. This is consistent with the view that lytic and latent pathways of gene expression in neurones diverge from each other during acute ganglionic infection (Margolis *et al.*, 1992; Speck & Simmons, 1992). The efficient silencing of the HCMV MIEP in the context of HSV-1 latency is well established (Arthur *et al.*, 2001; Balliet *et al.*, 2007; Scarpini *et al.*, 2001; Smith *et al.*, 2000) thus, the observation that there is no significant change in the average number of marked cells from days 30 (mean 87.3±9) to 150 p.i. (mean 83.9±10.2) (*P*=0.98) suggests that HCMV MIEP Cre expression marks a stable population of neurones that survived the initial infection. At present, we have no direct evidence that this population of marked cells represents the total reservoir of neurones containing latent HSV DNA. Nonetheless, our observations that the number of *β*-Gal-positive cells exceeds by ninefold the number of LAT-positive cells detected by *in situ* hybridization, together with previous reports demonstrating that only approximately 1/3 of all neurones containing HSV DNA express detectable levels of LATs (Maggioncalda *et al.*, 1996; Mehta *et al.*, 1995) suggests that HCMV MIEP-driven Cre expression is likely to have marked the majority of latently infected cells. Of significance, recent data using reporter mice in studies of HSV latency have revealed that the average viral copy number per cell marked with a WT HSV-1 CMV IE Cre construct is 38 genomes per cell (Wakim *et al.*, 2008), which is similar to estimates from single-cell analyses (Sawtell *et al.*, 1998; Chen *et al.*, 2002). These data provide additional evidence of the high efficiency of cell marking achievable with Cre expressing viral constructs in reporter mice.

Expression of Cre recombinase from the LAT promoter resulted in a distinct kinetic of cell marking in comparison to that observed using the HCMV MIEP construct. Thus, only small numbers of neurones were marked at the acute stage of infection and there was a significant increase in the number of marked cells from the acute (day 5) to the latent time points (day 30, *P*=0.000013; day 70 *P*=0.000037) with stable numbers of *β*-Gal-positive cells being observed from days 30 (mean 84±8.5) to 70 p.i. (mean 82.4±8). The number of cells marked by HSV LAT Cre and HSV CMV Cre are similar during latency, suggesting that the majority of infected neurones experience LAT promoter activation during the first 30 days after infection. In contrast, expression of *LacZ* under LAT promoter control in a virus context has previously shown to mark relatively small numbers of latently infected neurones (Lachmann & Efstathiou, 1997). Thus, methodologies relying on direct assays of promoter activity at specific time points rather than historical measures, are likely to have underestimated the total proportion of cells that can experience promoter activity over a prolonged time frame.

Of particular interest was the observation that HSV ICP0 Cre was able to mark a stable population of neurones during latency. For the following reasons we consider it likely that these neurones represent cells that experienced activation of this IE promoter prior to the establishment of latency. (i) Previous *in vivo* studies examining the activity of the ICP0 promoter during latency using HSV recombinants encoding ICP0 promoter-reporter gene constructs resulted in the detection of few, if any reporter gene-positive neurones during latency (Lachmann *et al.*, 1999; Shimeld *et al.*, 2001; Thompson *et al.*, 2003). These data support the view that the ICP0 promoter is effectively silenced during latency. Therefore, the marking of a large reservoir of latently infected cells in this system is most likely due to transient activation of the ICP0 promoter in a specific subpopulation of cells during latency establishment rather than a result of ongoing ICP0 promoter activity during established latency. (ii) HSV ICP0 Cre marked an average of 30.1 and 28.1 *LacZ*-positive neurones per ganglion at days 30 and 65, respectively, which constitutes approximately 1/3 of the total latent reservoir as defined by the number of neurones marked by the HSV CMV or HSV LAT Cre recombinants. (iii) In the unlikely event that this marking is due to low-level transcription during latency it is necessary to point out that this expression starts very early in latency, all cells are marked by day 30, and it is restricted to a stable subpopulation of infected cells.

This stability of cell marking implies the existence of two populations of latently infected cells that had either experienced or not, prior ICP0-mediated reporter gene activation.

The low efficiency of cell marking by the HSV gC Cre recombinant during acute infection was unexpected since this virus has no detectable phenotype either *in vitro* or *in vivo*. Therefore, the failure to observe large numbers of *β*-Gal-positive cells within ganglia at day 5 is unlikely to be a consequence of virus attenuation. A more likely explanation is that virus-induced host cell shut off and cell death precluded reporter gene activation at this acute time point, resulting in an underestimate of the number of infected cells. Thus, the late kinetics of Cre recombinase expression may prove to be incompatible with efficient cell marking during lytic infection as a consequence of virus-induced cytopathic effects. This view is supported by the marked reduction in the efficiency of this recombinant to induce reporter gene expression following infection of the SUA reporter cell line (Rinaldi *et al.*, 1999) in comparison to the ICP0 and CMV Cre recombinants (data not shown).

Previous studies on the frequency of spontaneous *in vivo* reactivation have estimated that reactivation occurs in approximately 1 out of every 50–90 000 latently infected neurones or 1 reactivating neurone per 10–17 ganglia (Feldman *et al.*, 2002; Sawtell, 2003). In the current study, the frequency of *β*-Gal-positive neurones revealed by using the HSV gC Cre virus is 1.9–2 per ganglion during latency, which is at least 10-fold higher than the expected frequency of spontaneous reactivation. One possible explanation for this high frequency of marking by the HSV gC Cre recombinant is that the small, but stable, population of *β*-Gal-positive neurones observed during latency constitute cells that have survived late gene expression, possibly as a result of non-cytolytic CD8 T-mediated abrogation of replication (Sheridan *et al.*, 2007; Simmons & Tscharke, 1992).

Mechanistically, the most widely accepted view of latency establishment is that it is the result of a failure of IE gene activation (Efstathiou & Preston, 2005; Garcia-Blanco & Cullen, 1991; Knipe & Cliffe, 2008). It is therefore of interest that the reporter mouse system identified a cohort of latently infected cells that had experienced prior ICP0 promoter activation since ICP0 is a major transcriptional activator that plays an important role in the initiation of lytic cycle replication and reactivation (reviewed by Everett, 2000). As there is increasing evidence that HSV utilizes post-transcriptional regulatory strategies (Thompson *et al.*, 2003; Umbach *et al.*, 2008) a key issue will now be to determine whether the ICP0 promoter mediated Cre expression accurately reflects ICP0 protein expression during neuronal infection *in vivo*. Nonetheless, previous studies utilizing an *in vitro* neuronal latency model system have suggested that ICP0 gene expression can precede latency establishment raising the possibility that neurones can tolerate some degree of lytic gene expression prior to the establishment of latency (Arthur *et al.*, 2001). Furthermore, there is evidence to suggest that the efficient establishment of HSV-1 latency is dependent on ICP0 expression (Wilcox *et al.*, 1990). In conjunction with the data reported in the current study it seems likely that HSV can follow at least two pathways into latency: one that is preceded by ICP0 promoter activation and another that follows a default pathway with no prior evidence of IE promoter activation. It will therefore be of considerable interest to determine whether the cell marking observed with the ICP0 promoter can be extended to other IE or E virus promoters and to determine the biological consequences of such promoter activation. Since only small numbers of latently infected cells are competent for virus reactivation, it will now be possible to determine whether populations of latently infected cells that have experienced different degrees of virus promoter activation prior to the establishment of latency represent cells with an altered propensity for reactivation. In addition, the marking of different subsets of latently infected cells using reporter mice offers a potentially powerful system for the isolation and biochemical characterization of latently infected neuronal subsets.
